# Ability to Cycle Despite Severe Freezing of Gait in Atypical Parkinsonism in Fahr's Syndrome

**DOI:** 10.1002/mds.23794

**Published:** 2011-05-28

**Authors:** Maria Stamelou, Maja Kojovic, Mark J Edwards, Kailash P Bhatia

**Affiliations:** 1 Sobell Department of Motor Neuroscience and Movement Disorders, UCL Institute of NeurologyLondon, United Kingdom; 2 Department of Neurology, Philipps UniversityMarburg, Germany

The retained ability to cycle despite freezing of gait (FOG) has been reported to be typical of patients with PD,^1^, ^2^ and the “bicycle sign” (i.e., the loss of the ability to cycle) has been suggested as a red flag indicative of atypical parkinsonism.^3^ However, we present here a patient with Fahr's syndrome and severe FOG, but a remarkably preserved ability to cycle 7 years after disease onset. Fahr's syndrome encompasses a group of neurodegenerative disorders associated with calcification of the basal ganglia, cerebellum, and other brain regions.^4^ Parkinsonism with FOG and early falls are part of the clinical phenotype of this disorder.^5^

This 57-year-old patient developed difficulty walking with falls at the age of 53, followed swiftly by stuttering, erectile dysfunction, and urinary urgency. There was no relevant family history. On examination, he had hypomimia, festinant speech, echolalia, and scanning dysarthria. There was bilateral, but asymmetrical, bradykinesia and rigidity, intermittent rest tremor and bilateral postural arm tremor, dysmetria, and dysdiadochokinesia. He had marked FOG and tended to festinate backward on the pull test (see Video, Segment 1). The Mini–Mental State Examination was 25/30. A CT brain scan showed widespread calcification within the basal ganglia and dentate nuclei consistent with Fahr's syndrome. Extensive investigation showed no other abnormalities, and a dopamine transporter (DAT) scan was normal. He was treated with levodopa (l-dopa) with a mild improvement of his symptoms. The patient reported that despite the marked FOG, he could get around his local village by cycling with no difficulty (see Video, Segment 2).

Here, we report a patient with atypical parkinsonism and severe FOG who, nonetheless, has a negative bicycle sign, which has been suggested to distinguish patients with PD from atypical parkinsonism. This case suggests that this sign should be used with some caution in this regard. It remains unclear why, in some patients, there is a dissociation between severe difficulty with generating leg movements while walking, manifesting as FOG, and preserved ability to generate leg movements while cycling, although some possible suggestions have been made.^2^ For example, the action of cycling might represent a type of an external pacing cue that helps to overcome freezing.^7^ Another notable aspect is that the speed of leg movements seems to improve when cycling. This is not simply an improvement related to the “on” state in PD patients, as this was also observed in our patient who had a normal DAT scan and little response to l-dopa. The improvement may be related to “paradoxical kinesia,” a brief, sudden period of mobility in response to stress or life-threatening events.^6^

The bicycle sign has been suggested as a new red flag for distinguishing PD from atypical parkinsonism.^3^ One issue with the previous report is that the atypical parkinsonian patients, taken together as a group, were significantly older and more impaired in terms of UPDRS, postural instability, and ataxia than PD patients. It is unclear whether patients in the earlier stages of atypical parkinsonian conditions also lose the ability to cycle. In conclusion, here, we demonstrate a patient with atypical parkinsonism and marked FOG resulting from Fahr's syndrome with a perfectly preserved ability to cycle 7 years after disease onset. We suggest that the bicycle sign should be used with caution as a red flag to distinguish PD from atypical parkinsonism.

## Legend to the Video

The first part of the video demonstrates the patient with Fahr's syndrome and severe freezing of gait, which improves when using a visual cue stick. The second part of the video shows the patient cycling without any difficulties.
